# The local GLP-1 system in the olfactory bulb is required for odor-evoked cephalic phase of insulin release in mice

**DOI:** 10.1016/j.molmet.2023.101738

**Published:** 2023-05-13

**Authors:** Mireia Montaner, Jessica Denom, Wanqing Jiang, Christophe Magnan, Stefan Trapp, Hirac Gurden

**Affiliations:** 1 Université de Paris Cité, Unit of Functional and Adaptive Biology (BFA), UMR 8251 CNRS, Paris, France; 2 Centre for Cardiovascular and Metabolic Neuroscience, Department of Neuroscience, Physiology & Pharmacology, UCL, London, UK

**Keywords:** Obesity, Olfactory bulb, Glucagon like Peptide-1, Cephalic phase insulin release, Foraging, M3 muscarinic receptors

## Abstract

**Objective:**

The olfactory bulb (OB) codes for sensory information and contributes to the control of energy metabolism by regulating foraging and cephalic phase responses. Mitral cells are the main output neurons of the OB. The glucagon-like peptide-1 (GLP-1)/GLP-1 receptor (GLP-1R) system in the OB (GLP-1^OB^) has been shown to be a major regulator of mitral cell activity but its function *in vivo* is unclear. Therefore, we investigated the role of GLP-1^OB^ in foraging behavior and odor-evoked Cephalic Phase Insulin Release (CPIR).

**Methods and results:**

By fluorescent labeling, we confirmed the presence of GLP-1 producing neurons and the expression of GLP-1R in the mouse OB. In response to food odor presentation, we collected blood, quantified plasma insulin by ELISA and showed the existence of an odor-evoked CPIR in lean mice but its absence in obese animals. Expression of shRNA against preproglucagon mRNA in the OB resulted in blunted CPIR in lean mice. Injecting Exendin (9-39), a GLP-1R antagonist, into the OB of lean mice also resulted in decreased CPIR. Since parasympathetic cholinergic input to the pancreas is known to be partly responsible for CPIR, we systemically administered the muscarinic M3 receptor antagonist 4-DAMP which resulted in a reduced odor-evoked CPIR. Finally, local injection of Exendin (9-39) in the OB extinguished olfactory foraging in lean mice whereas the injection of the GLP-1R agonist Exendin-4 rescued the loss of foraging behavior in obese mice.

**Conclusions:**

Our results demonstrate that GLP-1^OB^ controls olfactory foraging and is required for odor-evoked CPIR. We describe a new crucial brain function for GLP-1 and GLP-1R expressed within the brain.

## Highlights

• The GLP-1/GLP-1R system in the OB promotes odor-evoked cephalic phase insulin release.
• Obese mice lack odor-evoked CPIR.
• Antagonizing GLP-1R by exendin-9 in the OB of lean mice inhibits CPIR.
• Odor-evoked CPIR requires the activation of M3 muscarinic receptors.
• GLP-1R in the OB controls olfactory-driven foraging.

## 1 Introduction

Cephalic phase responses are preabsorptive anticipatory mechanisms, such as increased gastric secretion and intestinal motility, triggered by food-related stimuli acting on sensory receptors in the head and oropharynx [[1], [2], [3]]. They prepare the body for metabolic processing of the ingested nutrients [4,5]. Among these responses, the cephalic phase of insulin release (CPIR) [6] is essential for the early regulation of blood glucose following ingestion of nutrients [[7], [8], [9]] and is due to vagal activity [10,11] and muscarinic receptors [[12], [13], [14]] stimulating pancreatic beta cell activity.

In rodents, nocturnal foraging depends on olfactory cues [15] and smell sensitivity is increased in fasted states during foraging [16,17]. In humans, odors trigger cephalic responses such as changes in the quantity [18] and the quality of saliva [19] and gastric acid secretion [20].

The olfactory bulb (OB) is the first structure coding olfactory information in the mammalian brain [21]. It is involved in the regulation of energy homeostasis including cephalic phase responses, food intake and glucose and lipid utilization [[22], [23], [24]]. Mitral cells are the principal neurons of the OB. They receive direct synaptic inputs from olfactory neurons, which are responsible for (food) odor detection in the nose [25]. Mitral cells send projections and convey output information to the primary olfactory cortex and key limbic structures (piriform cortex, amygdala, entorhinal cortex, hypothalamus …) [26]. Mitral cells are densely connected to local OB interneurons such as granule cells and deep short axon cells [27,28]. These local interneurons shape odor-evoked mitral or tufted cell firing rates and synchrony in the process of sensory coding [29]. Interestingly, mitral cells express receptors for important metabolic hormones such as ghrelin, leptin, insulin and the incretin, glucagon-like peptide-1 (GLP-1) [30].

GLP-1 promotes glucose-stimulated insulin secretion by pancreatic beta cells and inhibition of food intake by the brain [31,32]. GLP-1 is also a neuropeptide acting locally in several brain areas [[33], [34], [35]]. Whilst most GLP-1 producing neurons are located in the Nucleus Tractus Solitarii (NTS) and have been demonstrated to be the main brain source of GLP-1 [32,33], GLP-1 mRNA has also been detected in the OB of rat by *in situ* hybridization [36] and GLP-1-producing deep short axon cells (also called preproglucagon cells, PPG cells) were identified in mice [37] and recently also in rat [38]. In addition, synaptic circuitry centered on the regulatory action of PPG cells on mitral cells was recently described in the OB [37,39]. While GLP-1–producing (PPG) neurons in the NTS mediate stress-induced hypophagia and large meal satiation [33] independently from the action of circulating GLP-1 [40], the role of the GLP-1/GLP-1R system in the OB (GLP-1^OB^) remains unknown *in vivo*.

In this context, we tested the hypothesis that GLP-1^OB^ might be a potent regulator of olfactory cue driven behavior and energy homeostasis during the cephalic phase. To this aim, we used pharmacological and genetic approaches targeted selectively to the OB to stimulate or block GLP-1^OB^ signaling in behavioral studies. We demonstrated that GLP-1^OB^ mediates olfactory foraging and is necessary for odor-evoked CPIR which is blocked by an M3 cholinergic antagonist.

## 2 Results

### 2.1 PPG neurons and GLP-1R + neurons are present locally in the mouse OB

**Figure 1:**
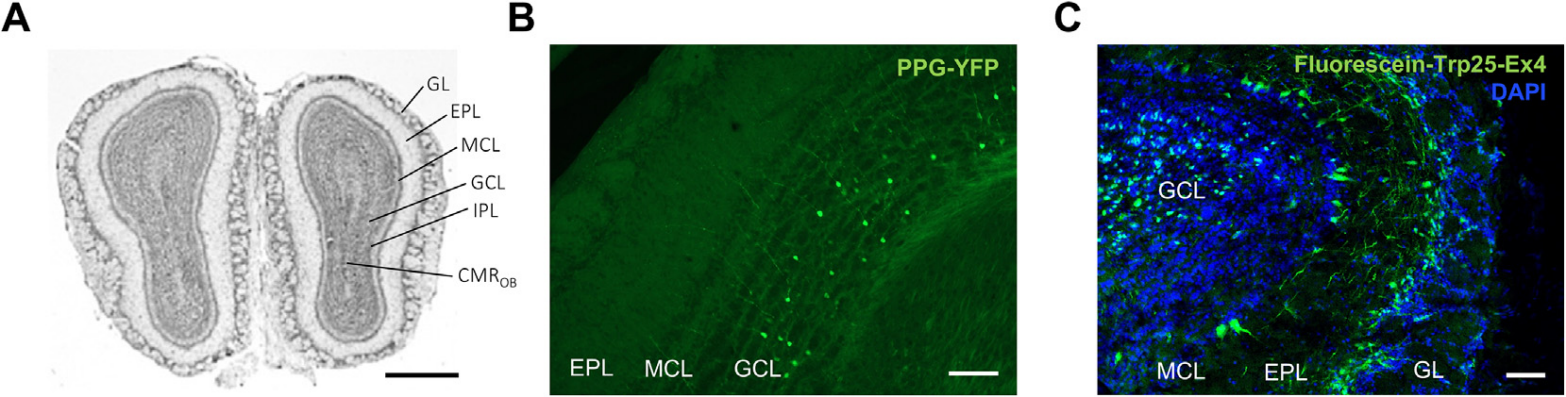
**Detection of PPG neurons and GLP-1 receptors in the mouse OB. A.** NeuN-stained mouse olfactory bulb section. GL: glomerular layer; EPL: external plexiform layer; MCL: mitral cell layer; ONL: olfactory nerve layer; GCL: granular cell layer; IPL: internal plexiform layer, CMR_OB_: bulbar part of the rostral migration stream. Scale bar = 500 μm (modified from Mounir Bendahmane thesis, https://theses.hal.science/tel-00737493). **B.** Representative photomicrograph of a PPG-YFP mouse OB showing YFP labelling of PPG cells in the GCL. GCL: granular cell layer; MCL: mitral cell layer; EPL: external plexiform layer; GLM: glomerular cell layer. Scale bar = 100 μm. **C.** Photomicrograph of representative OB section from a mouse injected with Fluorescein-Trp25-Exendin-4 into the OB. Fluorescence of Ex4 is shown in green, DAPI in blue. Scale bar = 100 μm.

We first observed PPG labeling of cellular populations in the OB of PPG-YFP mice. Within the granule cell layer (GCL; Figure 1A), we detected a population of PPG neurons as previously described [37] with their axon terminals reaching the mitral cell layer (MCL; Figure 1B). We next assessed the localization of GLP-1R in the OB. To this aim, GLP-1R-expressing OB cells were targeted using Fluorescein-Trp25-exendin-4, a fluorescently labeled Exendin-4. Fluorescein-Trp25-exendin-4 was internalized by neurons within the MCL, the GCL and the glomerular layer (GL) (Figure 1C). This result confirmed a previous study by Thiebaud et al. [37] that used a similar fluorescent Exendin-4 derivative and confirmed specificity of labelling in GLP-1R^−/−^ mice. Additionally, scattered stained cells were found within the GCL and the inner part of the GL.

### 2.2 GLP-1^OB^ promotes odor-induced CPIR

**Figure 2:**
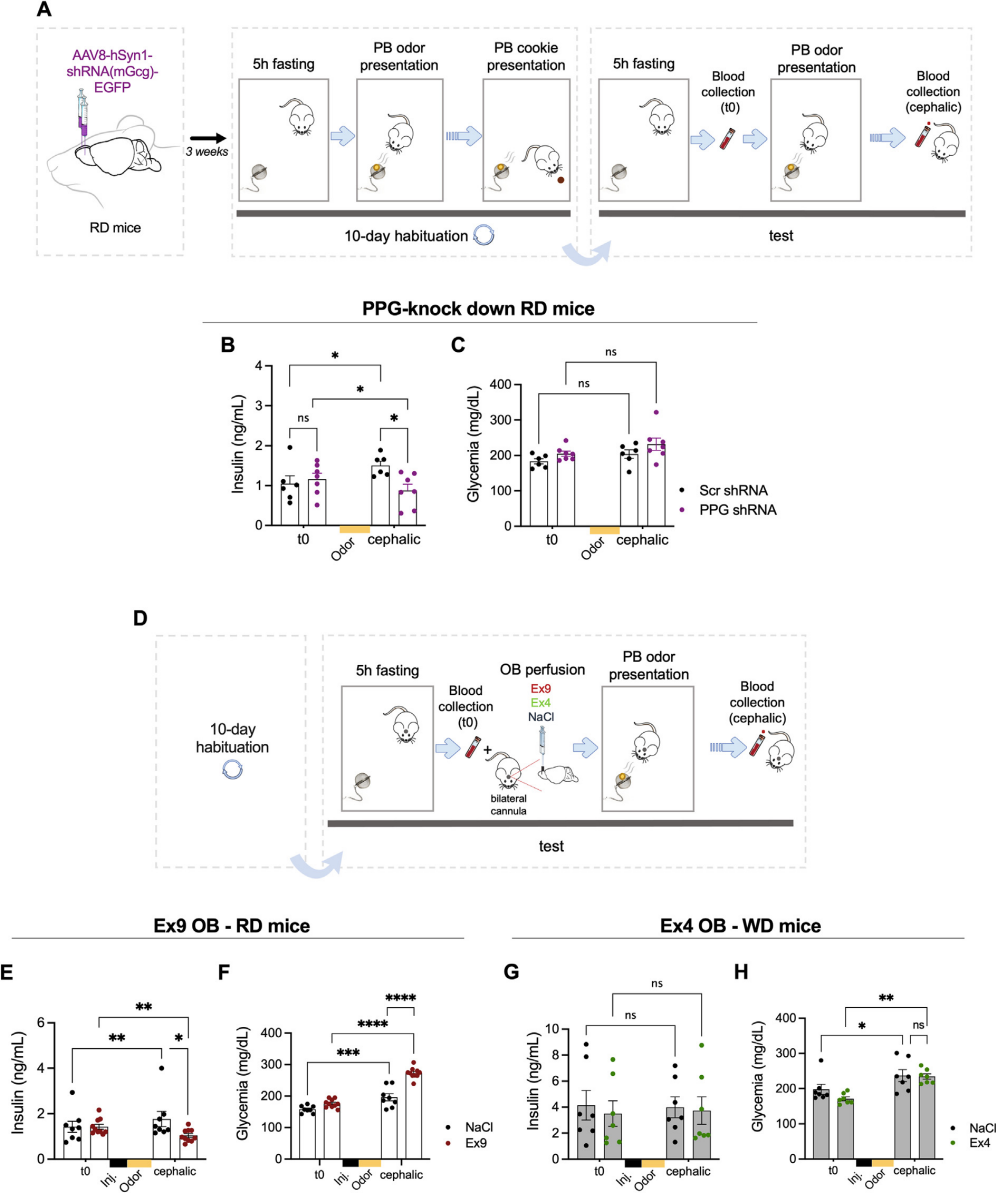
**The GLP-1 system in the OB promotes odor-induced CPIR. A**. Schematic illustration of viral delivery in the OB and subsequent protocol performed to assess odor-induced CPIR in PPG-shRNA RD mice. Yellow circle represents peanut butter (PB) odor inside a tea ball. Brown circle represents the PB-odorized cookie. **(B, C).** Plasma insulin and glucose levels at baseline (t0) and during the cephalic phase (induced by PB odor) in RD mice with PPG-shRNA (n = 6) or scrambled-shRNA (Scr shRNA, n = 7) in the OB. **D.** Schematic illustration of the protocol performed to assess the impact of Ex9 and Ex4 in the odor-induced CPIR in RD and WD mice. **(E, F)** Plasma insulin and glucose levels at baseline (t0) and during the cephalic phase induced by PB odor (cephalic) in RD mice with Ex9 injection into the OB compared to the same mice injected with NaCl (n = 7). The black square labelled “Inj.” represents the injection time of drugs in the OB, which precedes food odor presentation (orange square). **(G, H)** Plasma insulin and glucose levels at baseline (t0) and during the cephalic phase induced by PB odor in WD mice injected with Ex4 in the OB compared to the same mice injected with NaCl (n = 11). p-values were calculated by two-way ANOVA followed by post hoc Holm–Sidak test. Data are given as mean ± SEM. ∗p < 0.05; ∗∗p < 0.01; ∗∗∗p < 0.001; ns, not significant.

First, we explored the existence of odor-induced CPIR in Regular Diet fed (RD) mice and assessed whether PPG neurons in the OB play a role in this odor-evoked anticipatory insulin response. During the 10 days of habituation, peanut butter odor was coupled to the presentation of a peanut butter-odorized cookie to induce food odor learning (Figure 2A). On the test day, insulinemia and glycemia were quantified in response to food odor. Control RD mice expressing virally-delivered control-shRNA in the OB showed a significant early increase of insulinemia following peanut butter odor exposure (Figure 2B). In contrast, no odor-induced CPIR was observed in RD mice expressing virally-delivered PPG-shRNA, knocking down GLP-1 expression, in the OB (Figure 2B). Glycemia remained stable during the test in both groups (Figure 2C).

Then, we performed the same odor-induced CPIR experiment on RD mice and Western Diet fed (WD) mice (Figure 2D, see body composition of RD and WD mice in Supplementary Fig. 1). We assessed whether GLP-1^OB^ plays a key role in odor-induced CPIR in RD and WD mice. Analyzing different metabolic backgrounds enabled us to check whether WD is linked to a deficient odor-induced CPIR and whether GLP-1^OB^ could be a putative rescue for metabolic dysfunction in obesity. In response to peanut butter odor exposure, RD mice presented an early insulin release which was blocked by acute injection of Exendin (9-39) in the OB to block GLP-1R (Figure 2E) in accordance with the PPG-shRNA results. In contrast to RD mice, WD mice did not present an odor-induced CPIR (Figure 2G) and an OB injection of Exendin-4 could not reverse this loss. Unexpectedly, glycemia was increased in RD mice during the cephalic phase induced by peanut butter odor exposure in the NaCl-injected controls and even further after Exendin (9-39) delivery in the OB (Figure 2F). Since mice were connected to an injection catheter in a small arena where they were free to move but at a close distance of the Hamilton syringe, we think that this restriction could have caused stress and increased glycemia. In contrast, glycemia remained stable in the cephalic phase induced by peanut butter odor presentation after Exendin-4 delivery compared to the NaCl-injected WD group (Figure 2H). Overall, there was no correlation between glycemia and CPIR in the RD nor the WD group under control conditions (Supplementary Fig. 2).

Altogether, these results suggest that odor-induced CPIR is controlled by PPG neurons in the OB via the activation of local GLP-1R signaling.

### 2.3 Odor-induced CPIR is blocked by the M3 muscarinic receptor antagonist 4-DAMP

**Figure 3:**
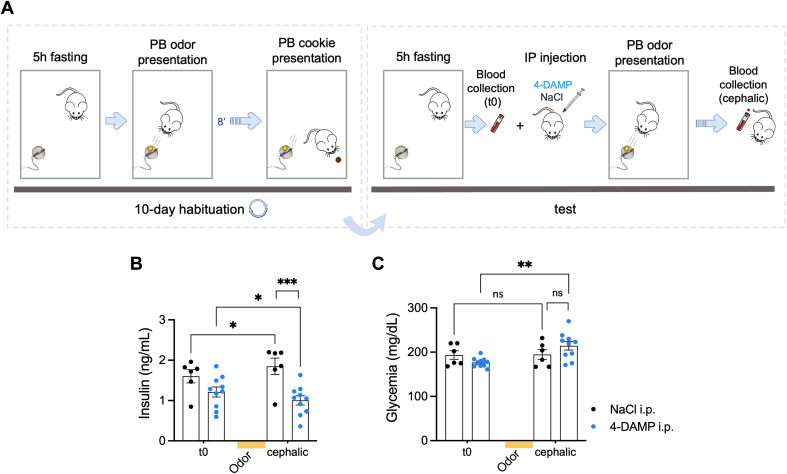
**Odor-induced CPIR is blocked by the M3 muscarinicreceptorantagonist 4-DAMP. A**. Schematic illustration of the protocol performed to assess the impact of 4-DAMP on odor-induced CPIR in RD mice. Yellow circle represents PB odor inside a tea ball. Brown circle represents the PB-odorized cookie. (**B, C**) Plasma insulin and glucose levels before (t0) and after (cephalic phase induction by PB odor) i. p. injection of 4-DAMP (n = 10) or NaCl (n = 6) in RD mice. p-values were calculated by two-way ANOVA followed by post hoc Holm–Sidak test. Data are given as mean ± SEM. ∗p < 0.05; ∗∗p < 0.01; ∗∗∗p < 0.001; ns, not significant.

Acetylcholine stimulates insulin secretion via activation of M3 muscarinic receptors [41]. To test whether they are involved in odor-induced CPIR, mice were injected intraperitoneally (i. p.) with the M3 receptor antagonist 4-DAMP before odor presentation (Figure 3A). As expected for a vagally-mediated effect, insulinemia was decreased in mice treated with 4-DAMP after peanut butter odor presentation, whereas the control NaCl group showed an intact CPIR (Figure 3B). Glycemia was increased in 4-DAMP-treated mice after odor exposure while no significant changes were found in the control group (Figure 3C). Finally, food odor-induced CPIR did not impact glycemia (Supplementary Fig. 2).

### 2.4 Olfactory detection is modulated by GLP-1R pharmacology in the OB of RD and WD mice

**Figure 4:**
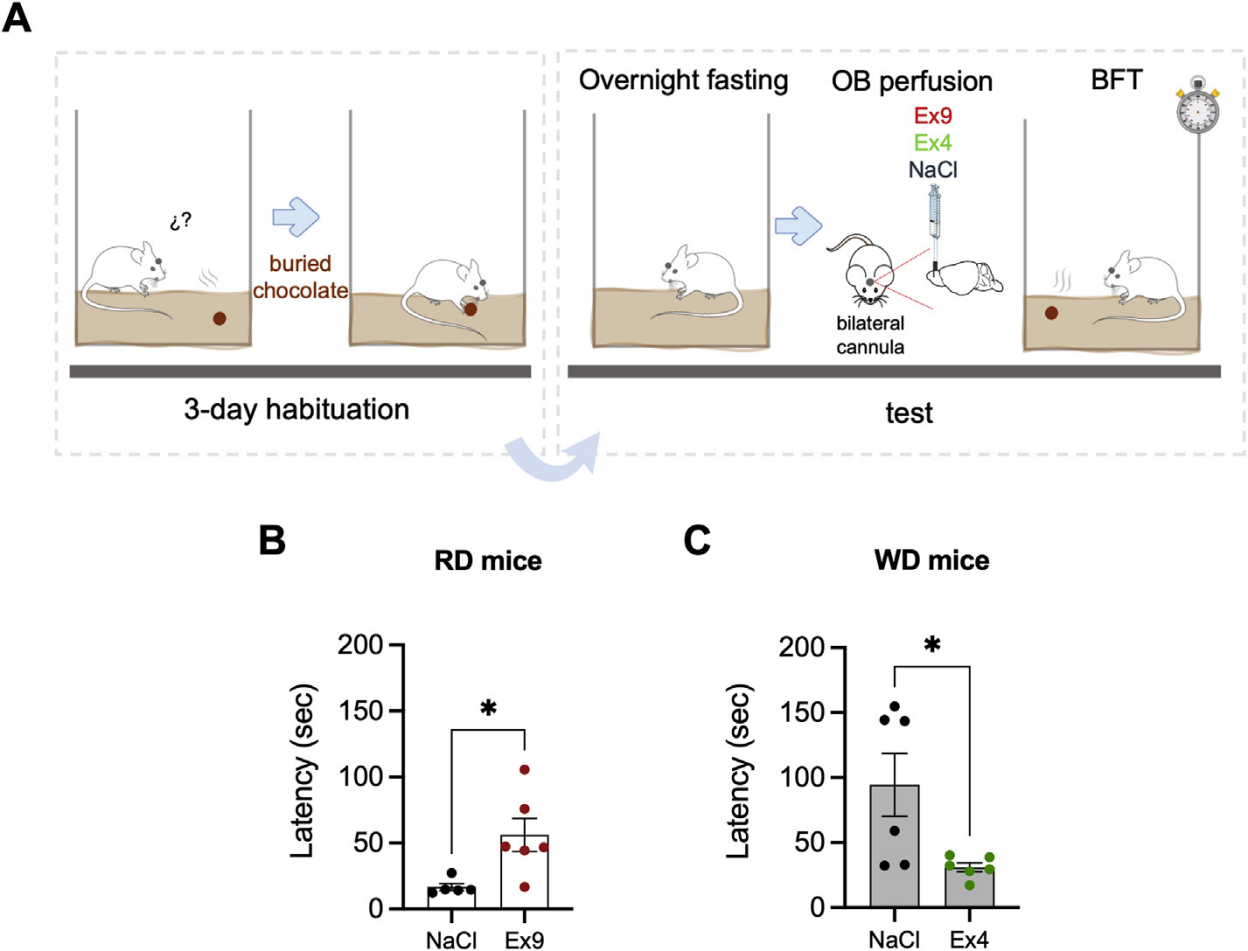
**Up- and down-regulation of olfactory detection by local GLP-1R pharmacology in the OB. A**. Schematic illustration of the buried food test (BFT) combined with pharmacological injections (Ex4, Ex9) in the OB of RD and WD mice. **B**. Latency to retrieve a hidden piece of chocolate after Ex9 injection in the OB for RD mice (n = 5–6/group). **C**. Latency to retrieve a hidden piece of chocolate after Ex4 injection in the OB for WD mice (n = 6/group). p-values were calculated by an unpaired Student's t test. Data are given as mean ± SEM. ∗p < 0.05; ∗∗p < 0.01; ∗∗∗p < 0.001; ns, not significant.

We next assessed the possible relationship between food odor detection and GLP-1R signaling in the OB. To this end, we performed a Buried Food Test on overnight fasted mice. The Buried Food Test is a standard behavioral approach which depends on rodents’ natural tendency to use olfactory cues for foraging [42,43]. Prior to the Buried Food Test, cannulated WD and RD mice underwent Ex4 and Ex9 injections in the OB, respectively (Figure 4A). NaCl-injected RD mice presented normal olfaction and optimal foraging time. The injection of Ex9 in the OB of RD mice significantly increased the latency to uncover a hidden piece of chocolate compared to controls (Figure 4B). WD mice, instead, presented a reduced ability to find the buried piece of chocolate, which reflected a significant decrease of olfactory sensitivity caused by the obesogenic diet, as reported previously [44]. Interestingly, the injection of Ex4 into the OB of obese mice corrected their reduced olfactory sensitivity compared to the NaCl-injected WD group (Figure 4C). Together, these findings suggest that GLP-1Rs in the OB are essential for the control of olfactory foraging.

## 3 Discussion

We demonstrated that GLP-1^OB^, consisting of an OB source of GLP-1 and OB GLP-1 receptors, is necessary for odor-induced CPIR and involves M3 muscarinic receptor activation. We also demonstrated that GLP-1^OB^-dependent CPIR is impaired in obese mice. In addition to this effect on insulinemia, we further showed that GLP-1^OB^ is crucial for olfactory foraging behavior, suggesting that OB-derived GLP-1 modulates the acuity of olfactory perception.

For the brain, food consists of complex multisensory signals to process [45]. Since food intake triggers a major change in energy homeostasis [46], anticipatory responses initiated in the cephalic phase are induced by sensory inputs and are essential to ensure the preparation of the body to the incoming nutrients [47]. Among these cephalic responses, the CPIR reaches a peak 5 ± 3 min after the presentation of the food stimulus [4] and lasts for around 10 min in humans. Although a role of odors and/or the OB was demonstrated in the regulation of hypothalamic network activity [48] and peripheral metabolic processing in the liver [49] and adipose tissue [50] in rodents, our study is the first to demonstrate that a food odor triggers CPIR in lean mice and also that odor-evoked CPIR is absent in obese mice. Indeed, failure of CPIR in obese mice could be due to difficulties in perceiving (food) odors [22,44,51] or to an impaired link between the olfactory system and the network leading to parasympathetic activity through the hypothalamic circuit [52]. An intact sense of smell is an absolute requirement for efficient foraging behavior at fasted state for rodents which perform foraging primarily at night. In order to navigate toward a food source, mice use food odor cues as a sensory guide [15] with mitral cells in the OB dynamically tracking the scent trail [53]. Here, we showed that activating GLP-1 receptors in the OB by local Ex4 injection caused a behavioral rescue of olfactory foraging in obese mice. In contrast, inhibiting GLP-1Rs with Ex9 infusion in the OB of lean mice extinguished olfactory foraging (i.e. mimicked an obese phenotype). This indicates that the GLP-1^OB^ system is a functional link between olfactory physiology (olfactory foraging) and preparatory metabolic processing to food intake (CPIR) expected to occur by the end of foraging.

Two lines of our results demonstrate a control of odor-evoked CPIR by a local source of GLP-1 within the OB. Firstly, local expression of PPG-shRNA knocking down GLP-1 expression in the OB inhibits CPIR. PPG neurons within the OB are locally projecting short axon cells or granule cells [37], that would deliver GLP-1 locally only to GLP-1Rs, e.g. on mitral cells. Secondly local injection of Ex9 into the OB inhibits CPIR. Both of these results point at GLP-1R activation within the OB as the source of odor-evoked CPIR. Bulbar GLP-1Rs would be primarily accessible to locally released GLP-1 from bulbar PPG neurons. Blood GLP-1 as a source for bulbar GLP-1R activation in our experiments seems rather unlikely, because mice are food-deprived, in a preabsorptive state during the odor-evoked CPIR test, thus with low circulating GLP-1, very unlikely to penetrate the OB and activate GLP-1R in the OB. Moreover, the half-life of GLP-1 is of the order of a few minutes in the blood ([31]; see also [34]) which also limits its access to the OB. Finally, projections from brainstem PPG neurons would be another hypothetical source of GLP-1 for OB GLP-1Rs. However, no such projections have been reported to date, and the aforementioned result that not only Ex9, but also local OB PPG-shRNA mRNA blocks the CPIR, argues against this source of GLP-1 for the odor-evoked CPIR.

Blood insulin levels are a direct and solid readout of CPIR. Although PPG knockdown and Ex9 injection in the OB convergently and similarly decreased CPIR, Ex9 injection resulted in an increase of concomitant glycemia while PPG knockdown did not. We hypothesize that this difference in blood glucose levels is related to the experimental difference between these two conditions. Mice are likely to undergo stress during acute drug injection by restrained movements due to catheter connections to the Hamilton syringe. In support of this notion, not only Ex9, but also saline significantly increased glycemia in these experiments. However, the additional difference between saline and Ex9 remains puzzling. In contrast, shRNA was injected 3 weeks before the CPIR test and mice were freely moving during the CPIR experiment. Plotting CPIR versus glycemia in control, drug-free conditions confirmed that there is no significant correlation between these two metabolic parameters. Therefore, our data on odor-evoked CPIR and glycemia are in line with the repeated demonstration that CPIR does not impact concomitant glycemia [14,54].

In rodents, vagal activity from the brain to the pancreas was shown to elicit CPIR by stimulating pancreatic beta cells [7,55]. In humans, administration of atropine, a global muscarinic receptor antagonist, blocks the CPIR [12,13]. Recently, the vagal input mediating CPIR was shown to be activated by the proinflammatory cytokine interleukin-1β (IL-1β) in healthy mice [14]. This study also showed that atropine was efficient to block IL-1β-induced CPIR. M3 muscarinic receptors are expressed by pancreatic beta cells and play a key role in maintaining proper insulin release [41]. In the present study, we demonstrated that odor-evoked CPIR is inhibited by systemic M3 receptor block further identifying muscarinic signaling as the effector of odor-evoked CPIR.

Whilst it is well established that olfactory and other sensory cues can trigger CPIR, and that the efferent vagal nerve is the essential common pathway to the pancreas (e.g. [56]), the circuitry from the olfactory sensory neurons via the olfactory bulb and cortex to the dorsal vagal complex in the lower brainstem has not yet been studied in depth. Bilateral communications between the OB and the hypothalamus have been described [57] and a direct activation of AgRP neurons in the arcuate nucleus by olfactory stimulation was recorded [58]. Since vagal activity can be controlled via several descending hypothalamic pathways, including the paraventricular nucleus [59], to the dorsal motor vagus nucleus, we postulate that the hypothalamus is very likely implicated in the intermediate network. Our findings here should provide the impetus and some of the tools to elucidate the anatomo-functional properties of this OB to pancreas circuitry in detail, in order to enhance understanding of the influence of the olfactory system on metabolic homeostasis.

Although clearly demonstrated in several mammalian species [55,60,61], CPIR has been extensively debated for many years and its relevance remains uncertain in humans, with very recent reviews based on metanalysis still divided [[3], [4], [5]]. In addition, Pullicin et al. [6] reviewed mechanistic and methodological issues underlying intra- and inter-study variability. Noteworthy, although meal presentation of food, i.e. combined visual and olfactory stimuli, is effective in triggering CPIR in healthy patients [62,63], it failed to do so in obese patients [64]. Thus, the lack of odor-evoked CPIR in our obese mice is consistent with the impaired cephalic phase insulin release observed in human obesity.

Food odor, in our case peanut butter odor, is a preparatory and predictive physiological signal during the cephalic phase before the prandial phase. Whilst our results confirm this general notion, our study also raises the question of which aspects of the odor, its nature (food versus neutral), its hedonic valence (pleasant versus unpleasant) or its metabolic significance (association to either high fat, high sucrose or high protein in the food) are crucial for this response. A comprehensive answer to these questions in follow-up studies should further cement the importance of these cephalic responses and generate ideas about its translatability for the treatment of metabolic disease.

In urban life, CPIR is very likely to occur when queuing, hungry, to buy lunch. In this context, an incremental series of sensory inputs stimulate the brain, starting with odor cues, then visual identification and ends with taste probing. Thus, odors are the very first excitatory signals arising from the orofacial area to activate the brain-pancreas axis. Since GLP-1R mRNA [65] is present in the human OB, and we have shown that Ex4 injection into the OB of obese mice rescued olfactory function, an interesting follow-up from our study would be to consider whether intrabulbar GLP-1 activity and subsequent CPIR could be enhanced by intranasal GLP-1 or Ex4 inhalation in obese patients. One promising study showed that treating T2D patients with application of nasal GLP-1 compound bypasses side effects such as nausea and vomiting of subcutaneous injections of GLP-1 agonists and reduces glycemia [66]. Indeed, intranasal injection of hormones is an efficient way to load the brain with these peptides via the olfactory bulb and the cerebrospinal fluid, with low concentrations of detected molecules in the blood stream [67].

Finally, it is interesting to consider that other common endocrine and neuronal signaling neuropeptides, such as somatostatin (SST), cholecystokinin (CCK) and vasoactive-intestinal-peptide (VIP), are also expressed by OB cells in mice. These peptides were shown to play a major role in the regulation of OB circuits in lean mice. Deletion of SST-Receptor-2 [68] as well as the inhibition of VIP [69] in the OB have deleterious effects on olfactory detection and discrimination behaviors. SST, VIP and CCK [70] are also complex regulators of mitral cell output activity. Our data are in line with these results and further explore the role of GLP-1^OB^ in the context of obesity. GLP-1^OB^ has already been shown to be a potent regulator of OB microcircuits [39]. Here, we add a new functional role of GLP-1^OB^ in the control of olfactory foraging in lean and obese mice. Furthermore, we demonstrate that GLP-1 in the OB is a neuropeptide that is essential for odor-evoked CPIR, thus functionally linking food sensory cues to pancreatic beta cell function in the preabsorptive state in mice.

## 4 Material and methods

### 4.1 Animals and diets

All animal studies in France were performed with approval of the “Buffon-Université Paris Cité” Ethics Committee (#7637, French Ministry of Research). Experiments conducted in the United Kingdom were performed in accordance with the UK Animals (Scientific Procedures) Act 1986, and experimental protocols were approved by the UCL Animal Welfare and Ethical Review Body (Bloomsbury Campus). In Paris 6-week-old C57BL/6 J male mice were purchased (Janvier Labs, Le Genest Saint Isle, France) and fed ad libitum with a mixed high-fat/high-sucrose diet (HF230 Diet 5317 kcal/kg, Safe, Augy, France) and regular chow (A04 Diet, 2791 kCal/kg, Safe, Augy, France) diet throughout the whole study. Age-matched controls were fed with regular chow diet ad libitum. In London, PPG-YFP [71] mice were bred in-house on a C57BL/6 J background and fed a regular chow diet (Teklad 2018 or 7912, Envigo) ad libitum. In both locations experimental mice were housed individually in cages in a room maintained at 22 ± 1 °C on a 12-h/12-h light–dark (L/D) schedule. All mice were accustomed to daily handling.

### 4.2 OB cell labeling

#### 4.2.1 YFP-immunoreactivity in GLP-1 neurons

PPG-YFP mice were deeply anesthetized with pentobarbital (200 mg/kg, i. p.) and transcardially perfused with heparinised (500 Units/l) phosphate-buffered saline followed by phosphate-buffered 4% paraformaldehyde (PFA). Brains were excised and post-fixed for 24 h in the same fixative. Subsequently, brains were transferred into PBS containing 30% sucrose and sectioned coronally at 30 μm thickness on a cryostat. Cryostat sections were processed free-floating for immunofluorescence labelling. Olfactory bulb sections were washed 3 × 5 min in PBS followed by 40min of blocking in PBS containing 0.3% Triton X-100, 10% goat serum, 1% bovine albumin. Primary antibody polyclonal chicken anti-GFP (Abcam #ab13970) was added at 1:1000 and incubated over night at 4 °C on a shaker. After 5 × 5 min washes in PBS, goat anti-chicken IgG Alexa Fluor 488 conjugate (1:500; Thermofisher #A-11039) was added in PBS containing 0.3% Triton X-100, 10% goat serum, 1% bovine albumin and incubated for 2 h in the dark on a shaker at room temperature. Subsequently, sections were washed 5 × 5 min in PBS, mounted on superfrost slides, dried and coverslipped with Vectashield.

#### 4.2.2 Fluorescein-Trp25-Exendin-4 distribution

Fluorescein-Trp25-Exendin-4 (0.5 μg/μl; Eurogentec) was injected bilaterally into the OB of cannulated RD and WD mice at a rate of 0.125 μl/min for 4 min. 90 min post-injection, mice were transcardially perfused with 4% PFA as described in *4.2.1*. After cryoprotection, the OB was cut into 18 μm-thick sections using a cryostat (Leica Biosystems). Brain slices were collected on microscope slides (SuperFrost Ultra Plus™ GOLD) and mounted with mounting media (VECTASHIELD® with DAPI). Image acquisition was performed with a confocal microscope (Zeiss LSM980 Airyscan2).

### 4.3 Metabolic phenotyping

Body weight was measured weekly throughout the study. Body mass composition was assessed using an Echo Medical Systems EchoMRI 100 (EchoMRI, Houston, TX, USA) at week 16. RD mice >40 g were excluded from all the procedures assessed in this work.

### 4.4 Surgical and stereotactic procedures

#### 4.4.1 Implantation of a guide cannula in the OB

A double stainless steel guide cannula (26 gauge in diameter, Plastic One, USA) was implanted through stereotactic procedures (AP: +4.8 mm from bregma, ML: ±1 mm and DV: 1.7 mm from brain surface) into the OB of mice under isoflurane anesthesia (1.5%, Isoflo, Abbott Laboratories Ltd, UK). Each side of the OB was thus implanted with one cannula barrel. To fix the guide cannula onto the skull, a plastic screw and dental cement (Super-Bond Universal Kit, Sun Medical and Unifast Trad) were used. A dummy was placed in the guide cannula to assure its patency and a reversible cap was screwed in the guide cannula to protect its tip. Mice were placed on a heated pad until they recovered from anesthesia.

#### 4.4.2 Viral delivery of shRNA in the OB

Mice were anesthetized with isoflurane as described in 4.4.1 and placed in a stereotaxic frame. Bilateral injections of an AAV expressing PPG-shRNA (AAV8-hSyn1-shRNA (mGcg)-EGFP) or scrambled control-shRNA (AAV8-hSyn1-shRNA (Non-Silencing)-EGFP) (Viral Vector Facility, Neuroscience Center Zuerich, Switzerland) were performed at a rate of 0.125 μl/min for 4 min (AP: +4.8 mm from bregma, ML: ±1 mm and DV: 1.7 mm from brain surface). The incision was sutured with Coated VICRYL suture (Ethicon) and mice were placed on a heated pad until they recovered from anesthesia.

### 4.5 Behavioral studies

#### 4.5.1 Odor-induced CPIR

Mice underwent 10 consecutive days of habituation prior to the test day. They were placed into experimental cages equipped with a tea ball hung in its lid and fasted for 5 h every day. Peanut-butter is a palatable and rewarding food commonly used in chemical senses studies [72,73]. During the habituation (days 1–10), a Peanut Butter (peanut butter liquid drops, the hut group, UK) -odorized filter paper was placed into the tea ball and mice were allowed to smell it for 8 min. Then, one piece of peanut butter-aromatized cookie per mouse was placed in a visible random location in the cage to trigger food odor learning. Three groups of mice underwent the test: i) OB-cannulated mice (RD and WD); ii) PPG-shRNA RD mice and iii) naive RD mice. On the test day (day 11), blood was collected from the tail vein (t0) and subsequently, RD- and WD-cannulated mice received bilateral OB injections of the GLP-1R antagonist Exendin (9-39) (Ex9; 25 μg/μl; Sigma–Aldrich), the agonist Exendin-4 (Ex4; 0.5 μg/μl; Sigma–Aldrich) or NaCl 0.9% for 4 min (0,125 μl/min). Injections were performed 13 min before odor presentation in awake behaving mice. 4-DAMP-injected RD mice instead underwent an i. p. injection of 4-DAMP (3 μg/kg), a selective antagonist of muscarinic M3 receptor, 10 min prior to odor presentation. After the first intense olfactory exploration of the tea ball by sniffing (1–2 min), blood was collected from the tail vein (cephalic phase). Blood cells were removed by centrifugation and plasma insulin was assayed with a wide-range Ultra Sensitive Mouse Insulin ELISA Kit (catalog no. 90080; Crystal Chem, Inc.).

#### 4.5.2 Olfactory foraging: buried food test

Single-housed cannulated WD and RD mice underwent two consecutive days (day 1 and day 2) of odorized-food familiarization (piece of chocolate, 1 g). On day 3, mice were fasted overnight. The test was performed on day 4 in the morning. Ex4 in WD mice and Ex9 in RD mice were infused in the OB 13 min prior to the behavior test. The test cage contained 5 cm of fresh bedding and was equipped with a video camera. A piece of chocolate was buried 2 cm beneath the surface of the bedding in a random location. Latency time (sec) was defined as the amount of time between the introduction of the mouse to the cage and the retrieval of the little piece of chocolate after foraging. Only mice that found the cookie were included in the statistics. Latency was manually counted using the Kinovea video tool (open platform, https://www.kinovea.org/).

### 4.6 Statistical methods

Data are given as means ± standard error of the mean (SEM). Statistical analysis was performed using unpaired Student's t test or two-way ANOVA after normality was assessed by a Shapiro–Wilk test. ANOVA tests were followed by two-by-two comparisons using Holm–Sidak post hoc test (GraphPad Software, La Jolla, CA, USA). Differences were considered significant at p < 0.05.

## Author contributions

MM, JD, WJ: data recordings and analysis. MM, CM, ST, HG: study design, writing original draft. HG: Supervision of the study.

## Funding

This work was supported by Research grant SFD-2019 France to CM and HG, the Medical Research Council UK (MR/N02589X/1 to ST) and a grant from the European Foundation for the Study of Diabetes Germany (Merck Sharpe Dohme grant to ST). Additional support was obtained from the Cities Partnership program UCL to ST, CM and HG. MM is supported by a “Université Paris Cité IdEx” PhD fellowship and a EUR GENE, G.E.N.E. Graduate School fellowship. WJ is supported by a UCL ORS scholarship and a CSC scholarship from the Chinese Government.

## Declaration of Competing Interest

The authors declare that they have no known competing financial interests or personal relationships that could have appeared to influence the work reported in this paper.

## Data Availability

Data will be made available on request.
