# Supplementary figures and images for: The local GLP-1 system in the olfactory bulb is required for odor-evoked cephalic phase of insulin release in mice
Source: Mol Metab. 2023 May 13;73:101738. doi: 10.1016/j.molmet.2023.101738 (PMC10212752; doi:10.1016/j.molmet.2023.101738)

**A**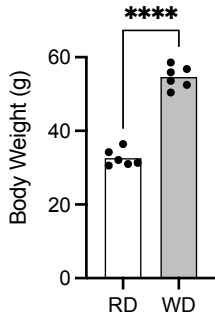**B**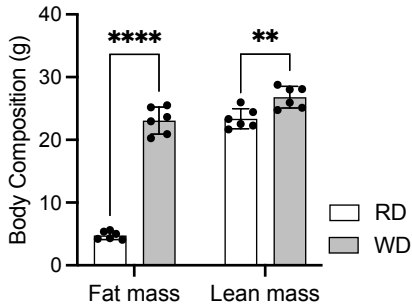**C**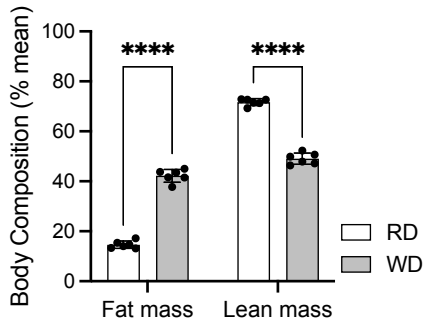

Supplement: Multimedia component 1 [file mmc1.pdf]

**A**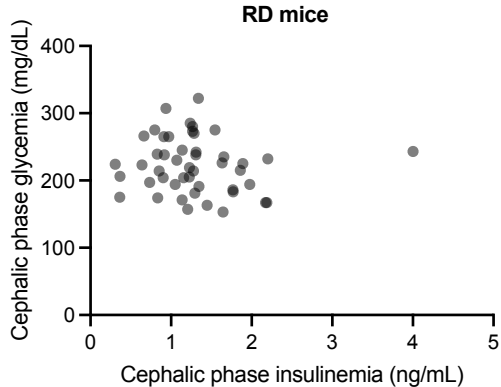**B**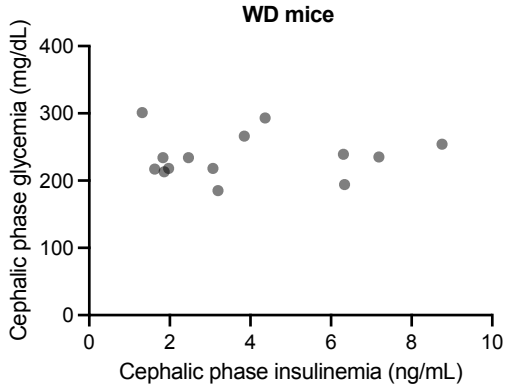

Supplement: Multimedia component 2 [file mmc2.pdf]
